# The Impact of Nutrition and Health Claims on Consumer Perceptions and Portion Size Selection: Results from a Nationally Representative Survey

**DOI:** 10.3390/nu10050656

**Published:** 2018-05-22

**Authors:** Tony Benson, Fiona Lavelle, Tamara Bucher, Amanda McCloat, Elaine Mooney, Bernadette Egan, Clare E. Collins, Moira Dean

**Affiliations:** 1 Institute for Global Food Security, School of Biological Sciences, Queen’s University Belfast, Belfast BT9 5AG, UK; t.benson@qub.ac.uk (T.B.); flavelle01@qub.ac.uk (F.L.); 2 School of Health Sciences, Faculty of Health and Medicine, The University of Newcastle, Callaghan, NSW 2308, Australia; tamara.bucher@newcastle.edu.au (T.B.); clare.collins@newcastle.edu.au (C.E.C.); 3 Priority Research Centre for Physical Activity and Nutrition, The University of Newcastle, Callaghan, NSW 2308, Australia; 4 Department of Home Economics, St. Angela’s College, F91 C634 Sligo, Ireland; amccloat@stangelas.nuigalway.ie (A.M.); emooney@stangelas.nuigalway.ie (E.M.); 5 Food, Consumer Behaviour and Health Research Centre, University of Surrey, Guildford, GU2 7XH, UK; m.egan@surrey.ac.uk

**Keywords:** nutrition claims, health claims, portion size, perceptions, health halo, food labelling, consumer, nudging

## Abstract

Nutrition and health claims on foods can help consumers make healthier food choices. However, claims may have a ‘halo’ effect, influencing consumer perceptions of foods and increasing consumption. Evidence for these effects are typically demonstrated in experiments with small samples, limiting generalisability. The current study aimed to overcome this limitation through the use of a nationally representative survey. In a cross-sectional survey of 1039 adults across the island of Ireland, respondents were presented with three different claims (nutrition claim = “Low in fat”; health claim = “With plant sterols. Proven to lower cholesterol”; satiety claim = “Fuller for longer”) on four different foods (cereal, soup, lasagne, and yoghurt). Participants answered questions on perceived healthiness, tastiness, and fillingness of the products with different claims and also selected a portion size they would consume. Claims influenced fillingness perceptions of some of the foods. However, there was little influence of claims on tastiness or healthiness perceptions or the portion size selected. Psychological factors such as consumers’ familiarity with foods carrying claims and belief in the claims were the most consistent predictors of perceptions and portion size selection. Future research should identify additional consumer factors that may moderate the relationships between claims, perceptions, and consumption.

## 1. Introduction

Obesity is a recognised risk factor for multiple chronic diseases including type 2 diabetes, coronary heart disease and some cancers [1,2,3]. Despite the known risks, rates of overweight and obesity have increased [4], with recent projections suggesting that over one in five adults worldwide will be obese by 2025 [5]. Obesity is a major public health issue on the Island of Ireland (IoI; includes Northern Ireland (NI) and the Republic of Ireland (ROI)), with 34% of adults overweight and a further 26% classified as obese in NI (UK) [6], and 39% overweight and 23% obese in ROI [7]. 

The aetiology of obesity is complex, with numerous causal factors such as genetics, lack of physical activity, and social and psychological factors [8,9]. At a basic level, one common cause of obesity is diet, with an individual gaining weight through excess energy intake relative to energy expenditure [8]. Obesity action plans have highlighted the need to encourage and support individuals to make healthier choices, particularly in relation to food, eating, and diet [10,11]. One method of helping individuals to make healthier food choices is through labelling and nutrition information on food and drink.

A nutrition claim is any claim on food products which states, suggests or implies that a food has beneficial nutritional properties due to the energy it provides or does not provide or the nutrients or other substances it contains or does not contain [12]. A health claim is any message conveyed in text or images that states, suggests or implies that a relationship exists between a food category, a food or one of its constituents and health [12]. Within the UK and Ireland, claims relating to fat such as ‘low in fat’ are the most prevalent nutrition claims, while claims relating to the digestive system and cholesterol or the cardiovascular system are the most common health claims [13,14]. One of the main purposes of Nutrition and Health Claims (NHCs) is to inform food purchasing and consumption decisions, and this in turn may help individuals to achieve a healthy, balanced diet [15]. 

While NHCs can be useful consumer aids, evidence has shown that they can have a ‘halo’ effect, influencing consumers’ perceptions of products carrying claims. Theory from cognitive psychology and information processing may explain this ‘halo’ effect. The spreading-activation theory of processing [16] suggests that memory is organized in networks containing interlinked knowledge and concepts. When knowledge is primed (in this case through a NHC), other concepts and links in our information network are then activated. For example, when one sees a ‘no cholesterol’ claim on packaging, further links in our knowledge network are then activated such as low-fat, health, and reduction of heart disease [17]. Therefore the presence of a claim may lead a consumer to generalize and infer, and in this example lead to a product with a ‘no cholesterol’ claim being viewed as healthy regardless of its nutrient content. Wang and colleagues [18] found that a health symbol increased healthiness perceptions but did not affect tastiness perceptions of snacks in their adolescent sample. A health claim placed on bread, yoghurt, and pork was also found to impact consumers’ healthiness, naturalness, attractiveness, and tastiness perceptions [19]. Similarly, nutrition claims have been found to affect healthiness perceptions of cookies [20]. However, in an experiment which examined perceptions and consumption of cereal, a “smart choices” claim was found to have no impact on healthiness or tastiness perceptions [21]. These differences may be due to the type of product carrying the claim and their typical nutritional properties. 

Numerous studies have investigated the effects of NHCs on perceptions, yet there is a lack of studies which have examined the effects of labelling on consumption [21]. This is important as it has been found that claims, which could help healthy eating, may paradoxically lead to increased consumption or energy intake [22]. Indeed, a recent systematic review and meta-analysis found only six studies (all experiments) that examined the impact of NHCs on consumption [23]. Results from these studies were mixed but overall it was found that NHCs increase purchasing and/or consumption of food and drink products [23]. 

In addition to NHCs, other factors which have been found to influence consumers’ perceptions and intake or food selection include gender [24,25], age [25], socioeconomic status [26], education [27], familiarity with the food [28] and psychological variables including knowledge [29], cognitive restraint, uncontrolled eating, emotional eating, and general health interest [28]. 

While some studies have examined the effects of NHCs on perceptions and consumption, these have typically been experiments with relatively small numbers of participants, limiting the generalisability of results. Indeed, two recent systematic reviews highlight the need for further studies in this area [23,30]. The dearth of studies using a representative sample must be addressed given that NHCs on products have the potential to be seen by and impact upon the behaviour and portion size selection of all consumers in the population. Furthermore, few studies of NHCs have been conducted on the IoI. Previous studies have shown differences between countries relating to NHCs and labelling. For example, 65% of individuals in Ireland check nutrition information always or occasionally, compared with 52% in the UK, 50% in Sweden, and 63% in France [31]. There are also differences between countries with regards to health claims and foods and attitudes to the compatibility of foods carrying NHCs as functional foods [32]. The current study aims to address these limitations by using a representative survey to understand the effects of NHCs on consumer perceptions and portion size selection on the IoI.

The hypotheses of the study are:

1. The presence of an NHC will impact consumers’ perceived healthiness, perceived tastiness, and perceived fillingness of a product. The direction of the effect will depend on the product-claim combination.
2. The presence of an NHC will impact consumers’ portion size selection. The direction of the effect will depend on the product-claim combination.

## 2. Materials and Methods 

### 2.1. Sampling

To ensure geographical representation, sampling points for the survey consisted of local government districts and urban/rural locations across IoI. National representativeness was achieved using quota sampling, with quotas applied for age (18–64 years old), sex, and socioeconomic status in both NI and ROI based on official census data. Those working or living in a household with anyone working in advertising, marketing, the food industry or a nutrition and diet-related area were excluded. In addition, those with severe food allergies or intolerances, vegans, vegetarians or pescetarians, and those who had never eaten any of the foods selected for examination in the survey were also excluded.

### 2.2. Procedure

Prior to data collection, the questionnaire was piloted with 12 individuals for clarity and timing, with minor amendments made to wording. Interviewers from a market research company called at households within areas selected and conducted interviews face-to-face using Computer Assisted Personal Interviewing (CAPI). Respondents answered questions as outlined below and were then shown a randomly selected product show card and answered perceptions questions followed by choosing a portion size that they would consume. Participants completed this in turn for each of the four foods. Interviews lasted approximately 25 min. Ethical approval was obtained from the Queen’s University Belfast School of Biological Sciences Research Ethics Committee and was in accordance with the Declaration of Helsinki. Respondents provided informed consent prior to completing the survey and did not receive an incentive or payment for their participation. 

### 2.3. Questionnaire

Following a review of the literature, items were created to measure factors previously found to impact consumption or portion size selection. These were included alongside other relevant sociodemographic, household, and psychological items to form the study questionnaire. 

#### 2.3.1. Food Packaging (NHCs)

**Figure 1:**
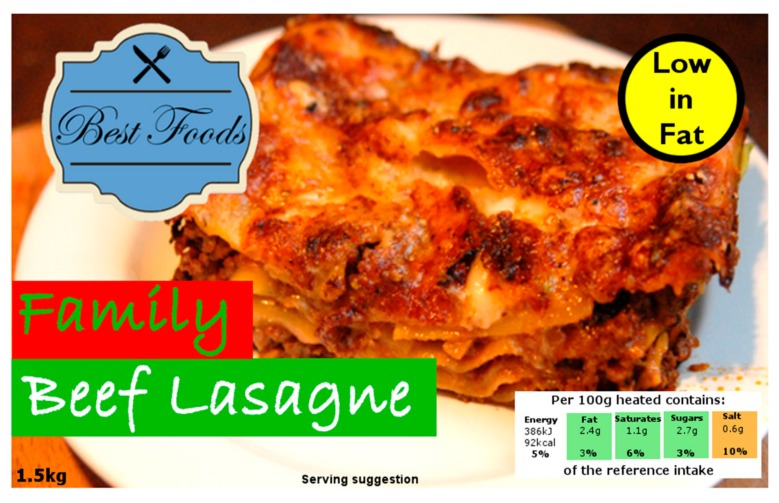
Example of food packaging with claims used in survey. Lasagne photograph from Leon Wilson (https://www.flickr.com/photos/chop/973369384)—CC BY 2.0 (https://creativecommons.org/licenses/by/2.0/). Modified to include labels and text.

To examine the impact of NHCs on perceptions and portion size selection, images of different food packages were created (see Figure 1 for example of product packaging used). In total, four foods were used, cereal (breakfast), soup (lunch), lasagne (dinner), and yoghurt (snack). These products were selected based on their amorphous (portionable) nature and availability of validated portion size photographs. In addition, as each product was required to carry each of the selected claims, it was important that each product-claim combination had ecological validity and was realistic.

Three NHCs were used; the nutrition claim ‘Low in fat’, the health claim ‘With plant sterols. Proven to lower cholesterol’, and the satiety claim ‘Fuller for longer’. These claims were taken from products sold in the UK and Ireland. In addition, the absence of a NHC (no claim) was also investigated. ‘Low in fat’ was chosen due to the prevalence of claims relating to fat in the UK and Ireland [13,14]. ‘With plant sterols. Proven to lower cholesterol’ was chosen as claims relating to cholesterol and heart health were common [13,14], and it was believed that these claims would be more suitable for the foods chosen than other common health claims such as those relating to digestion. ‘Fuller for longer’ is not currently an official registered nutrition or health claim, however, this has been previously used on products within the UK and Ireland. Furthermore, previous research which investigated satiety claims (claim only with no product) suggested that future research should examine the impact of satiety claims in the context of product packaging [33]. 

Generic branding was used across all products. For authenticity, product images were also labelled with an appropriate product weight and Guideline Daily Allowance (GDA) traffic lights nutrition summary label. The weight and GDA summary label (low fat version for each food) were the same within each product, regardless of the claim displayed. In total, there were 16 different possible show cards (4 meals × 4 claim types), with participants answering questions on a random allocation of four (each individual product with a different claim). For example, an individual participant might answer questions on cereal with no claim, soup with satiety claim, lasagne with health claim, and yoghurt with nutrition claim.

#### 2.3.2. Food Perceptions and Portion Size Selection

For each product, three items measured participants’ tastiness, healthiness, and fillingness perceptions on seven point scales: “Thinking about this product, on a scale of 1 to 7 where 1 means not tasty/not healthy/not filling at all and 7 means extremely tasty/healthy/filling, to what extent do you think this product is tasty/healthy/filling?”

**Figure 2:**
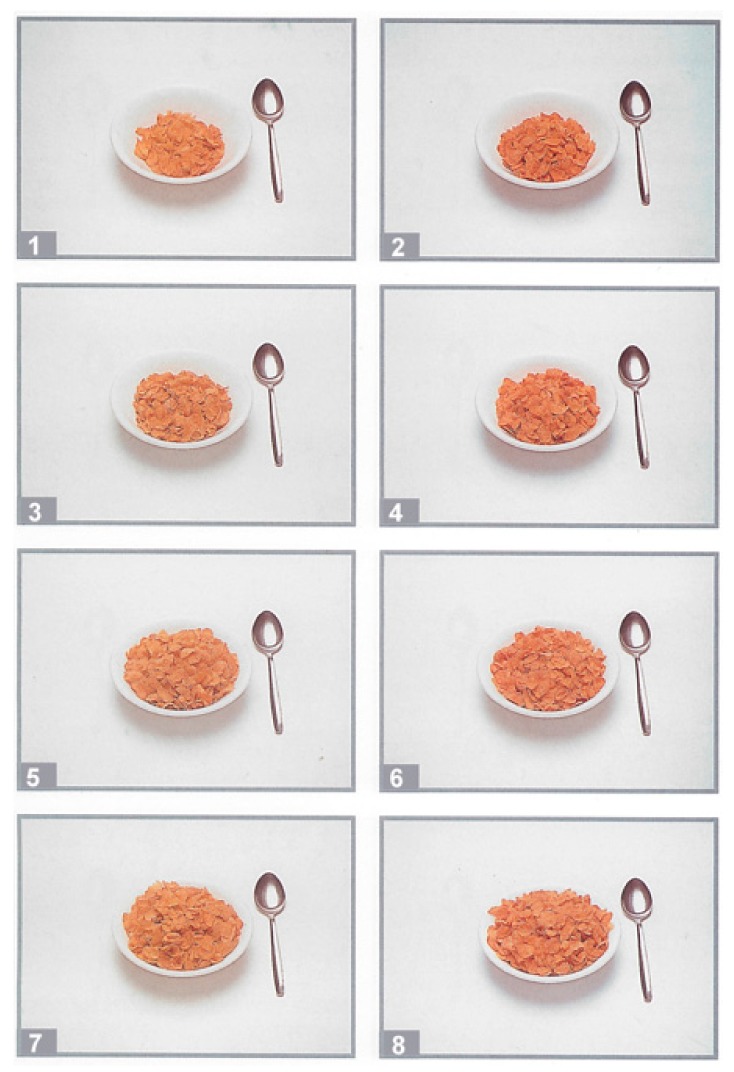
Example of portion size selection show card used in survey for cereal. Taken from validated food atlas [34].

For portion size selection, participants were asked “Imagine you are only having this specific product for breakfast/lunch/dinner/a snack. How much would you eat?” For each product, participants selected a portion from a show card which contained a series of eight portion size photographs (see Figure 2 for an example). Each series of portion size photographs was taken from a validated food atlas [34], with portions increasing in weight from the fifth through to the 95th percentile of portion size from a population diet and nutrition survey [35].

#### 2.3.3. Sociodemographic Measures

In addition to age, sex, and highest level of education, participants’ socioeconomic status was calculated based on the occupation status of the highest income earner in the household. Higher (ABC1) status included higher and intermediate managerial or professional as well as supervisory occupations. Lower (C2DE) status included skilled, semi-skilled or unskilled occupations as well as those unemployed.

Body Mass Index (BMI) was calculated using self-reported height and weight (weight in kilograms divided by square of height in metres). The World Health Organisation cut-offs were used to classify respondents to underweight (<18.50), normal weight (18.50–24.99), and overweight (>25).

#### 2.3.4. Psychological Measures

Current appetite status was measured using a seven-point scale for hunger (1 = not hungry at all, 7 = extremely hungry) and a similar scale for thirst (1 = not thirsty at all, 7 = extremely thirsty). These items were previously used in a portion size selection survey [28].

Attitudes towards the healthiness of foods were assessed using the General Health Interest (GHI) scale [36]. The scale consists of eight items measured from 1 (strongly disagree) to 7 (strongly agree), for example, “I am very particular about the healthiness of food I eat”. Negatively worded items were reverse scored and the mean of all summed items was used to provide a total GHI score, ranging from 1 to 7, with a higher score indicating greater health consciousness. Internal consistency, a measure of reliability, for the scale was good (α = 0.80) and in line with previous research [36,37].

Eating behaviour was measured using the revised Three-Factor Eating Questionnaire (TFEQ-R18) [38]. The TFEQ-R18 consists of three scales; cognitive restraint (six items such as “I deliberately take small helpings as a means of controlling my weight”), uncontrolled eating (nine items such as “Sometimes when I start eating, I just can’t seem to stop”), and emotional eating (three items such as “When I feel anxious, I find myself eating”). Participants indicated their agreement to the items using various four point scales such as 1 (definitely false) to 4 (definitely true). Items for each scale were then totaled, with a higher score indicating greater inclination for each type of eating behaviour. In line with previous research [39], internal consistency for the scales was good (cognitive restraint α = 0.76, uncontrolled eating α = 0.86, emotional eating α = 0.82). 

#### 2.3.5. Nutrition and Health Claim Knowledge

After rating their perceptions and choosing portion sizes, participants Subjective NHC knowledge (SNHCK) was assessed using three items adapted from Moorman and colleagues [40]. Participants were provided with definitions of nutrition and health claims and rated their self-perceived knowledge on statements such as “Compared to most people I am quite knowledgeable about nutrition and health claims”. Items used a scale of 1 (strongly disagree) to 5 (strongly agree). Items were summed to create a subjective knowledge score ranging from 3 to 15, with a higher score indicating greater subjective knowledge. Internal consistency for the scale was excellent (α = 0.90). Internal consistency for the original, similar scale was α = 0.88. 

Objective NHC knowledge (ONHCK) was measured using five items. Two of these items were adapted from previous research [41]: “The claim ‘iron contributes to normal cognitive function’ in other words means…” and “The claim ‘omega-3 fatty acids help to maintain a healthy cardiovascular system’ in other words means…”. The remaining three items were constructed based on European Union legislation [12] and the Nutrition and Health Claims Register [42]. All items had four possible answers, of which one answer was correct. Participants’ objective knowledge score was the number of correct answers provided, ranging from 0 to 5, with a higher score indicating greater objective knowledge.

#### 2.3.6. Other NHC and Food-Related Variables

The believability of the NHCs used in the survey was assessed by asking participants how much they believed each claim (“Low in Fat”; “Fuller for Longer”; “With plant sterols. Proven to lower cholesterol”). Believability in this instance refers to how much an individual believes that a claim is accurate or true. Responses were recorded on a scale from 1 (not believable at all) to 7 (extremely believable). Scores could therefore range from 3 to 21, with a higher score indicating greater belief in NHCs. A median split (score of 13) was used to classify respondents into those who did not believe in the claims used (below median) and those who believed in the claims used (median and above). 

Three items adapted from previous research [43] were used to measure motivation to process NHCs (MTPNHC), for example, “I pay attention to nutrition and health claims on food”. Items used a scale from 1 (strongly disagree) to 5 (strongly agree). Scores could therefore range from 3 to 15, with a higher score indicating greater motivation. Internal consistency for the scale was excellent (α = 0.92), similar to the original scale consistency of α = 0.94 [43].

Similar to a previous portion size selection survey [28], to measure familiarity with each of the four types of food examined in the survey (cereal, soup, lasagne, yoghurt) respondents were asked how often they eat each food. Respondents selected from one of the following options: “Never”, “Less than once a year”, “Once or twice a year”, “Every few months”, “Once or twice a month”, “Once a week”, “A couple of/few times a week”, and “Daily”.

To assess recall and understand if participants were attending to the claims presented, at the end of the survey respondents were asked to identify which NHCs they had seen on the photographs of packaging used in the survey. Six options were provided, three of which had been used in the survey, alongside three claims which had not been featured in the survey. Correct answers (the participant answered yes to having seen a claim that was used in the survey or no to having seen a claim that was not used in the survey) were scored as 1 and summed, meaning that participants could score a minimum of 0 and a maximum of 6, with a higher score indicating greater recall. 

### 2.4. Statistical Analysis

All data were analysed using IBM SPSS Statistics v22 (SPSS Inc., Chicago, IL, USA). Descriptive statistics (means (M), standard deviation (SD)) were used to explore the data. Analysis of Variance (ANOVAs) with Tukey’s HSD post-hoc tests were used to assess differences between the different nutrition and health claims on portion size selection, perceived healthiness, tastiness, and fillingness. A series of hierarchical multiple regression analyses were used to understand the relative contribution of NHCs (over and above possible covariates) to perceived healthiness, tastiness, and fillingness for the different foods across the claims groups and for portion size selection across all groups. 

## 3. Results

### 3.1. Participants

In total, 1039 participants aged 18–64 years old (M = 41.7, SD = 13.3) completed the survey (see Table 1 for sociodemographic details). Location, gender, age, and socioeconomic status are representative of the IoI. In terms of education, almost three in 10 (29.5%) were educated to university level. Respondents were more likely to believe in the NHCs featured in the survey (57%) and were slightly health conscious with a mean GHI score of 4.3 (maximum possible 7). Respondents believed they had relatively good knowledge of NHCs with a mean score of 10.23 (range 3 to 15). However, actual (objective) NHC knowledge was relatively low with a mean score of 2.32 from a maximum of 5. Individuals had motivation to process NHCs, with a mean motivation score of 9.86 (minimum possible 3, maximum possible 15). With regards to the recall of claims that were used in the survey, participants had a median score of 4 out of a possible 6 (minimum 0 and maximum 6), indicating general awareness of the NHCs used in the survey. 

### 3.2. Effects of NHCs on Perceptions

Results from the ANOVAs which examined tastiness and healthiness perceptions were not significant (not shown—all *p* > 0.13), indicating that participants perceived the tastiness and healthiness of the foods the same regardless of the claims on the food. In terms of fillingness, participants perceived lasagne and soup to be equally filling regardless of the claims on these foods. However, yoghurt with the fuller for longer claim was perceived to be more filling (M = 4.48, SD = 1.51) (scale: 1 not at all filling to 7 extremely filling) than yoghurt with the lowers cholesterol claim (M = 4.09, SD = 1.66) [F(3,1035) = 2.79, *p* = 0.04]. Furthermore, cereal with the lower cholesterol claim obtained a lower fillingness rating (M = 4.27, SD = 1.47) compared to all other claims (no claims M = 4.60, SD = 1.43; low fat M = 4.64, SD = 1.37; fuller for longer M = 4.66, SD = 1.43) [F(3,1035) = 4.54, *p* = 0.003]. 

Hierarchical regressions were used to examine the effects of NHCs after controlling for physiological, sociodemographic, and psychological factors (Table 2). The sociodemographic, psychological, and claim type factors explained 7% to 12% of the total variance in taste perceptions, 2% to 11% of the total variance in healthiness perceptions, and 3% to 11% of the total variance in fillingness perceptions. Results showed that psychological variables explained the largest amount of variance in perceptions of healthiness, taste, and fillingness (accounting for 2–9% of the variance). Overall, claims explained little variance in the prediction of perceptions over and above psychological and sociodemographic variables. Specifically, claims explained an extra 0.5% of variance for healthiness perceptions of cereal and between 0.6% and 1.7% of variance for fillingness perceptions of lasagne, cereal, and yoghurt.

In terms of tastiness, in the group of sociodemographic variables, education was the most consistent significant sociodemographic predictor (Table 2). Those with a lower education status perceived greater tastiness for soup and yoghurt. In addition, those who were less familiar with the foods perceived all the products to be tastier. Furthermore, those who believed in the claims also perceived the foods to be tastier. Finally, those with higher scores in uncontrolled eating perceived soup and lasagne to be tastier. However, the perceived tastiness was not affected by the type of claim shown on the packaging.

With regards to healthiness, those with a lower education status perceived greater healthiness for yoghurt, while older age predicted greater healthiness perceptions for lasagne (Table 3). As was found for tastiness, familiarity with the food and belief in claims were significant predictors of healthiness. Those who believed the claims and those who were less familiar with the foods perceived all foods to be healthier. In addition, when the satiety claim ‘Fuller for Longer’ was present, participants were more likely to perceive cereal as less healthy. 

Education was a significant sociodemographic predictor for fillingness; those with a lower education were more likely to perceive soup and cereal as more filling (Table 2). In the psychological variables group, those who were uncontrolled eaters were more likely to perceive soup and lasagne as filling. Again, food familiarity and belief in the claims were the strongest predictors, with the exception of lasagne and belief in claims for cereal. Greater belief in claims and less familiarity with the foods predicted a higher fillingness perception. Of the claims-related variables, the presence of the ‘fuller for longer’ claim was associated with a higher fillingess perception for lasagne but a lower fillingness perception for cereal. 

### 3.3. Effects of NHCs on Portion Size Selection

Results from the ANOVAs showed no differences between the types of claims seen on consumers’ portion size selections, with the exception of lasagne where there was a trend for those who saw lasagne with a NHC selecting smaller portions compared to those who saw lasagne with no claims (no claims M = 5.05, SD = 1.77; low fat M = 4.67, SD = 1.87; fuller for longer M = 4.64, SD = 1.95; lower cholesterol M = 4.82, SD = 1.77) [F(3,1035) = 4.54, *p* = 0.049].

Hierarchical multiple regressions were used to examine the impact of NHCs on portion size selection, after controlling for covariates. Across all hierarchical regressions (Table 3), current physiological status was controlled for in the initial step. Psychological variables for the model were added, explaining the largest amount of variance in all models (accounting for 9–14% of the variance). The addition of claim types significantly explained further variance only for lasagne (accounting for an additional 0.5% of the variance). 

In terms of sociodemographic factors, gender was a significant predictor for portion size selection, with men selecting larger portions than women (Table 3). Those with higher uncontrolled eating scores also chose larger portions. General Health Interest (GHI) was a significant predictor across all food types, with those with lower GHI selecting larger portion sizes. Additionally, those who were less familiar with the foods and those who believed the claims selected larger portion sizes. While having a claim influenced portion size selection of lasagne, the type of NHC did not affect portion size selection for any of the foods.

## 4. Discussion

The present study was the first to use a survey to understand the impact of NHCs on consumer portion size selection as well as perceived fillingness, healthiness, and tastiness of foods. Claims affected the fillingness perceptions of some foods, although overall relatively little of the variance in perceptions or portion size selection could be explained by NHCs. In particular, the satiety claim ‘fuller for longer’ affected the fillingness and healthiness perceptions of some of the foods. Psychological factors were more consistent predictors of perceptions and portion size selection. Specifically, familiarity with the food and believability in the NHCs that were present were the most consistent significant predictors of tastiness, healthiness, and fillingness perceptions. Familiarity and believability were also consistent predictors of portion size selection alongside gender, GHI, and uncontrolled eating. 

The effect of the ‘fuller for longer’ satiety claim on fillingness perceptions is perhaps unsurprising given the similar wording of ‘fuller’ in the claim and ‘fillingness’ in the question, as well as the links between satiety and fillingness [44]. The European Food Safety Authority (EFSA) states that claims relating to satiety or fullness should be supported by scientific evidence from human intervention studies. This evidence should show a change in appetite or satiety measurements, effects which remain after repeated consumption, and comparative claims with a control food [45]. The ‘fuller for longer’ claim is not authorized within the EU, nevertheless, these results suggest that the restrictive regulations in this area may be justified given the potential for an effect on the consumer. The ability of the ‘fuller for longer’ claim to influence perceptions but not portion size selection is perhaps due to an accurate understanding of satiety claims by consumers, who recognise the limited effects of such products with these claims [33]. 

While the ‘fuller for longer’ claim influenced healthiness perceptions of cereal, overall there was no impact of claims on how healthy participants believed the foods to be. Similarly, previously a ‘smart choices’ logo with a written claim was found to have no impact on tastiness or healthiness perceptions of cereal [21]. However, numerous studies have found an impact of claims on perceived healthiness [18,20,46]. Compared to the current study which used a single written claim on each food, these studies have used verbal, symbol only, or multiple claims together. The differences in foods used in studies may also explain different findings. It has been suggested that the inherent healthiness of the product carrying the claim has a stronger effect on perceived healthiness than the claim [47]. The differences in foods and types of claims used across studies highlights the heterogeneity in this field. Factors other than claims may have a greater influence on consumer perceptions, with NHCs explaining only 0–2% variance in perceptions in the current study. For example, education influenced perceptions (particularly tastiness and fillingness) of some of the foods. This may relate to previous research which has found that education is linked to interest in and use of labels [48,49]. In addition, it has been found that priming may depend on individual characteristics such as level of education [50]. This suggests that in the context of the spreading-activation theory of information processing [16], a NHC as a prime may lead to different network and memory activations depending on an individual’s education level or other personal characteristics. Factors which were not examined in the present study such as emotions, branding, food origin, production date, and type of processing may also explain greater variance than claims [47,51]. 

Similar to the findings relating to perceptions, claims explained little variance in portion size selection. As might be expected, hunger, gender, uncontrolled eating, and general health interest significantly predicted portion size selection. Familiarity with the foods carrying the claims and believability in NHCs were also significant predictors. Those who were less familiar with the foods and those who believed in the claims selected larger portion sizes. This negative relationship between familiarity and portion size selection has been found previously. Individuals select smaller portion sizes of more familiar foods due to expected satiation (with more familiar foods expected to be more filling and therefore smaller portions are selected) [52,53,54]. It is interesting that those who believed in the claims selected larger portions (and also viewed the products as tastier, healthier, and more filling). To our knowledge, no previous studies have examined the impact of believability of NHCs on perceptions or portion size selection. This finding suggests that personal or consumer characteristics are pertinent factors in the relationships between claims and consumption and claims and perceptions. This is in line with the conclusions of a recent systematic review and meta-analysis that consumer attributes may influence the strength of relationships between claims and their effects [23]. Future research should identify the most important consumer attributes that influence the effects of claims, and also explore the relative pathways and strength of these factors. This could in turn inform the development of interventions and education campaigns to target those who may be influenced by NHCs and select or consume larger portion sizes as a result. 

A strength of the present study was the use of a survey with a nationally representative sample, thereby improving generalisability of the findings. To our knowledge, this is the first study to use a representative sample to examine the impact of NHCs on portion size selection. Another strength was the use of previously established scales and items where possible in the questionnaire, enhancing the comparability and validity of the findings. For example, portion size selection photographs were from a validated food atlas [34] based on population consumption. However, it should be noted that some single items were used in the questionnaire and multiple items may have been more appropriate to measure some constructs. 

Limitations of the study include the use of a survey method which meant that portion size selection rather than consumption was adopted as an outcome measure. While this means that it cannot be concluded that individuals would entirely consume their selected portion, previous research suggests that individuals tend to “plate-clean” and consume meals in their entirety [55,56]. As expected, males selected larger portions than females in our study. This finding, alongside the previous use of this portion size selection method and similar uses [28,56], including a validated web buffet which found good agreement between portion size selection from photographs and actual consumption [57], supports the validity of the method used in the current study. A further limitation inherent to surveys is the use of self-report data. The topics explored in the questionnaire such as eating behaviours and portion size selection may be particularly susceptible to response bias. However, this is unlikely to have considerably affected the main focus of the study to compare perceptions and portion size selection in the presence of different claims. Response bias will likely have affected all of a participant’s portion size selections and therefore comparison between conditions is unlikely to have been affected. 

## 5. Conclusions

Nutrition and health claims affected perceptions of food but had little impact on portion size selection. Consumer characteristics such as familiarity with the food and believability in the claim may moderate the impact of NHCs on perceptions and portion size selection. 

## Figures and Tables

**Table 1:** Characteristics of sample.

		*n* (%)
	Total	1039 (100%)
Location	NI	328 (31.6)
	ROI	711 (68.4)
Gender	Male	485 (46.7)
	Female	554 (53.3)
Age	18–34	336 (32.3)
	35–49	396 (38.1)
	50–64	307 (29.5)
Socioeconomic status	ABC1	506 (48.7)
	C2DE	533 (51.3)
Education	Primary school or less	48 (4.6)
	Secondary school to age 15/16	167 (16.1)
	Secondary school to age 17/18	300 (28.9)
	Additional training	217 (20.9)
	Undergraduate	231 (22.2)
	Postgraduate	76 (7.3)
BMI	Underweight	21 (2.0)
	Normal weight	372 (35.8)
	Overweight/obese	389 (37.4)
	Refused/unknown	257 (24.7)
Belief in NHCs	Believed in claims selected	592 (57.0)
	Did not believe in claims selected	447 (43.0)
		***M (SD)***
TFEQ-R18 ^1^	Cognitive restraint	14.10 (3.6) ^2^
	Uncontrolled eating	19.90 (5.4) ^3^
	Emotional eating	6.60 (2.4) ^4^
GHI ^5^		4.3 (1.1)
Subjective NHC knowledge		10.20 ^6^
Objective NHC knowledge		2.32 ^7^
Motivation to process NHCs		9.86 ^8^
NHC recall		4.00 ^9^

^1^ Three Factor Eating Questionnaire—Revised 18; ^2^ Possible range 6 to 24; ^3^ Possible range 9 to 36; ^4^ Possible range 3 to 12; ^5^ General Health Interest with a possible range of 1 to 7; ^6^ Possible range 3 to 15; ^7^ Possible range 0 to 5; ^8^ Possible range 3 to 15; ^9^ Possible range 0 to 6.

**Table 2:** Explained adjusted variance and standardized coefficients (β) for each regression for perceptions after the addition of each step.

	Tastiness Soup	Tastiness Lasagne	Tastiness Cereal	Tastiness Yoghurt	Healthiness Soup	Healthiness Lasagne	Healthiness Cereal	Healthiness Yoghurt	Fillingness Soup	Fillingness Lasagne	Fillingness Cereal	Fillingness Yoghurt
	β	β	β	β	β	β	β	β	β	β	β	β
**Step 1: Physiological**												
**Adjusted R^2^**	0.003	0.009 *	0.02 ***	0.02 **	−0.002	0.02 ***	0.01 **	0.001	0.003	−0.002	0.010 **	0.001
Hunger	0.08	0.06	0.10 *	0.12 **	−0.01	0.13 **	0.07	0.04	−0.03	−0.03	0.04	0.01
Thirst	−0.13 **	−0.11 *	−0.08 *	−0.06	−0.02	−0.06	−0.06	0.01	0.04	−0.01	0.03	−0.02
**Step 2: Sociodemographic**												
**Adjusted R^2^**	0.008	0.02 *	0.02	0.03 *	−0.002	0.03 *	0.02	0.02 **	0.002	0.001	0.02	0.002
Age	−0.03	−0.02	0.04	−0.11 **	−0.001	0.08 *	−0.01	−0.02	0.02	−0.04	0.02	−0.03
Gender	0.02	−0.05	−0.05	0.02	0.03	−0.04	−0.04	−0.01	0.02	−0.001	−0.04	0.05
Socioeconomic status	−0.03	−0.07	0.01	−0.03	−0.03	−0.03	−0.01	−0.06	0.000	−0.03	0.01	0.004
Education	−0.12 **	−0.07	−0.06	−0.12 **	−0.08	−0.04	−0.07	−0.12 **	−0.09 *	−0.07	−0.11 **	−0.07
**Step 3: Psychological**												
**Adjusted R^2^**	0.07 ***	0.08 ***	0.12 ***	0.12 ***	0.02 ***	0.10 ***	0.11 ***	0.04 **	0.05 ***	0.02 **	0.10 ***	0.06 ***
Cognitive restraint	−0.07	−0.01	0.00	0.02	−0.02	0.05	0.02	0.07	0.05	0.04	0.08	0.06
Uncontrolled eating	0.15 **	0.15 **	0.04	0.07	0.15 **	0.03	0.06	0.06	0.13 *	0.11 *	−0.03	0.03
Emotional eating	−0.07	−0.06	−0.01	−0.10 *	−0.13 **	−0.05	−0.06	−0.10	−0.06	−0.06	−0.04	−0.06
General Health Interest	0.10 *	−0.09	0.02	0.08	0.05	−0.15 **	−0.07	−0.03	0.13 **	−0.01	−0.06	−0.01
Motivation to process	−0.06	0.11	−0.11	0.002	0.03	0.03	−0.05	−0.12 *	0.01	−0.14 *	0.01	0.05
Subjective NHCK	0.11 *	0.03	0.09	0.04	−0.01	0.01	0.06	0.10	−0.07	0.09	0.01	−0.01
Objective NHCK	−0.02	0.06	−0.12 **	0.04	0.002	−0.09 *	−0.12 **	−0.03	−0.06	0.09 *	−0.19 ***	−0.06
Believers vs. non-believers	0.08 *	0.11 **	0.26 ***	0.19 ***	0.13 **	0.11 **	0.25 ***	0.12 **	0.12 **	0.07	0.21 ***	0.17 ***
Familiarity with food	−0.21 ***	−0.14 ***	−0.12**	−0.20 ***	−0.08 *	−0.15 ***	−0.08 *	−0.07 **	−0.12 **	−0.05	−0.06	−0.12 **
**Step 4: Claims**												
**Adjusted R^2^**	0.07	0.09	0.12	0.12	0.02	0.09	0.11 *	0.04	0.05	0.03*	0.11 ***	0.06 *
Low fat	−0.003	0.02		−0.07	0.02	−0.03		−0.02	0.005	−0.003		−0.08
Fuller for longer		−0.05	−0.07	−0.002		−0.01	−0.10 *			0.09*	−0.15 ***	
Lowers cholesterol	0.01		−0.01		0.04		−0.03	−0.03	0.04		−0.02	0.03

Subjective NHCK = Subjective Nutrition and Health Claims Knowledge; Objective NHCK = Objective Nutrition and Health Claims Knowledge; * = *p* < 0.05; ** = *p* < 0.01; *** = *p* < 0.001.

**Table 3:** Explained adjusted variance and standardized coefficients (β) for each regression for portion size selection after the addition of each step.

	Soup Portion Size	Lasagne Portion Size	Cereal Portion Size	Yoghurt Portion Size
	β	β	β	β
**Step 1: Physiological**				
**Adjusted R^2^**	0.06 ***	0.04 ***	0.07 ***	0.05 ***
Hunger	0.12 **	0.06	0.10 **	0.12 **
Thirst	0.02	0.004	0.06	−0.04
**Step 2: Sociodemographic**				
**Adjusted R^2^**	0.10 ***	0.09 ***	0.13 ***	0.07 ***
Age	−0.004	−0.003	−0.03	0.02
Gender	−0.17 ***	−0.17 ***	−0.18 ***	−0.14 ***
Socioeconomic status	0.01	−0.004	0.005	−0.005
Education	−0.03	−0.03	−0.03	−0.03
**Step 3: Psychological**				
**Adjusted R^2^**	0.19 ***	0.21 ***	0.27 ***	0.19 ***
Cognitive restraint	0.007	−0.08 *	0.05	0.02
Uncontrolled eating	0.19 ***	0.21 ***	0.14 **	0.15 **
Emotional eating	−0.10 *	−0.09 *	−0.07	−0.10 *
General Health Interest	−0.12 **	−0.12 **	−0.26 ***	−0.14 ***
Motivation to process	−0.07	0.07	0.03	0.05
Subjective NHCK	0.14 **	0.03	0.09 *	0.07
Objective NHCK	−0.09 **	−0.03	−0.10 ***	−0.14 ***
Believers vs. non-believers	0.15 ***	0.17 ***	0.23 ***	0.20 ***
Familiarity with food	−0.07 **	−0.13 ***	−0.08 **	−0.13 ***
**Step 4: Claims**				
**Adjusted R^2^**	0.19	0.21 *	0.27	0.19
Low fat	0.009	−0.03	−0.04	−0.02
Fuller for longer	0.002	−0.03	−0.02	0.06
Lowers cholesterol	0.04	0.06	0.01	0.04

Subjective NHCK = Subjective Nutrition and Health Claims Knowledge; Objective NHCK = Objective Nutrition and Health Claims Knowledge; * = *p* < 0.05; ** = *p* < 0.01; *** = *p* < 0.001.
